# Pneumatosis intestinalis with complete remission: a case report

**DOI:** 10.1186/1757-1626-2-7079

**Published:** 2009-04-29

**Authors:** Aly Saber

**Affiliations:** 1 Department of general surgery, Port-Fouad General Hospital, Al-obour street- Port-Fouad, 11361, Egypt

## Abstract

**Introduction:**

Pneumatosis cystoides intestinalis is a rare disease characterized by presence of multilocular cysts in the gastrointestinal wall. Rarely, patients may experience symptoms secondary to the cysts. The pathogenesis of pneumatosis cystoides intestinalis is still unclear and many theories have been advocated to explain the exact origin. Complications occur in about 3% of cases and include obstruction, intussusception, volvulus, haemorrhage and intestinal perforation.

**Case presentation:**

The author reported a male patient aged 56 years presented to the emergency department with acute upper abdominal pain. Widespread variable sized serosal intestinal air cysts were seen at the first look involving long segment of jejunum and ileum. Perforated duodenal ulcer, as the cause of generalized peritonitis, was repaired with direct closure and omental patch. A second laparotomy, was done and exploration was systematically performed and denoted hugely distended stomach with cicatrisation at the site of previous closure of perforated duodenal ulcer and the whole length of small gut was completely free from the already described pneumatosis cystoides intestinalis.

**Conclusion:**

The pneumatosis cystoides intestinalis is a rare disease and suspicion of this disease process should be based on imaging and clinical finding. The therapy can be conservative or surgical in restricted situations.

## Introduction

Pneumatosis cystoides intestinalis is a rare disease characterized by presence of multilocular cysts in the gastrointestinal wall. Idiopathic and secondary forms of the disease can be distinguished [1]. In the primary idiopathic form, multiple thin-walled cysts develop in the submucosa or subserosa of the gut. Usually, this form has no associated symptoms, and the cysts may be found incidentally through radiography or endoscopy [2]. The secondary form (85% of cases) is associated with obstructive pulmonary disease, as well as with obstructive and necrotic gastrointestinal disease [1,2].

Primary pneumatosis is often asymptomatic. Rarely, patients may experience symptoms secondary to the cysts. Signs and symptoms include diarrhea, bloody stools, abdominal pain, abdominal distention, and constipation. The physical findings are usually unremarkable [3].

Pneumatosis cystoides intestinalis may be associated with bowel ischemia, perforation, and a high mortality rate. As a result, many authorities advocate an aggressive surgical approach in those patients [4].

The pathogenesis of pneumatosis cystoides intestinalis is still unclear and many theories have been advocated to explain the exact origin [5]-[7]. The mechanical theory, which is the most accepted explanation and suggests that gas under pressure, is forced into the bowel wall through a mucosal defect associated with trauma, surgery, endoscopy and bowel obstruction [5,7].

Second, there is the bacterial theory. In animal experiments, introduction of bacteria into the bowel wall by injection has been shown to cause PCI [8]. The pulmonary theory has been criticized as the assumption that gas travels from ruptured alveoli through the mediastinum into retroperitoneal space and finds its way along perivascular spaces through the mesentery into the bowel wall could not be proven convincingly [7].

Complications occur in about 3% of cases and include obstruction, intussusception, volvulus, haemorrhage and intestinal perforation [5,6].

## Case presentation

A fit and otherwise healthy male patient aged 56 years from the Asian part of Egypt, presented to the emergency department with acute upper abdominal pain with three hours duration. The patient was in agony with anxious look. Due to religious background, he did not drink alcoholic beverages. His body temperature was 37.7°C and pulse rate was 88 beats per minute. Abdominal examination revealed tenderness over the epigasrtic region and the right side of the abdomen with rigidity maximally detected all over the right abdomen.

Plain x-ray films detected free gas under the diaphragm and abdomino-pelvic ultrasonography demonstrated free fluid in the peritoneal cavity.

**Figure 1:**
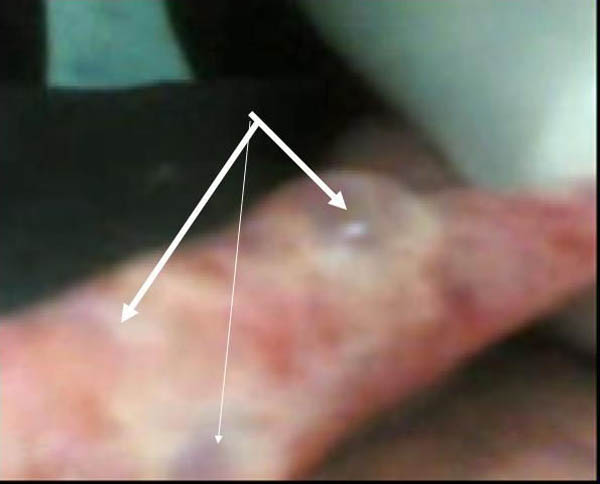
**A photograph showing widespread variable sized serosal intestinal air cysts involving long segment of jejunum and ileum**. All cysts were intact and were not incriminated as a cause of pneumoperitoneun or peritonitis.

After the routine work up and proper resuscitation, laparotomy was performed. Widespread variable sized serosal intestinal air cysts were seen at the first look involving long segment of jejunum and ileum. All cysts were intact and were not incriminated as a cause of pneumoperitoneun or peritonitis (Figure 1). Perforated duodenal ulcer, as the cause of generalized peritonitis, was repaired with direct closure and omental patch. Nothing was done for the intestinal air cysts. Metronidazol intravenous infusion was given two times daily.

The patient passed very smooth postoperative course and was followed up for the next eight months until the complaint of repeated vomiting was evident and food particles of eaten diets since more than two days were recognized in the vomitus.

Upper gastro-duodenal endoscope and barium meal study were performed and revealed gastric outlet obstruction. Computed axial tomography also confirmed the diagnosis and denied any other pathology.

**Figure 2:**
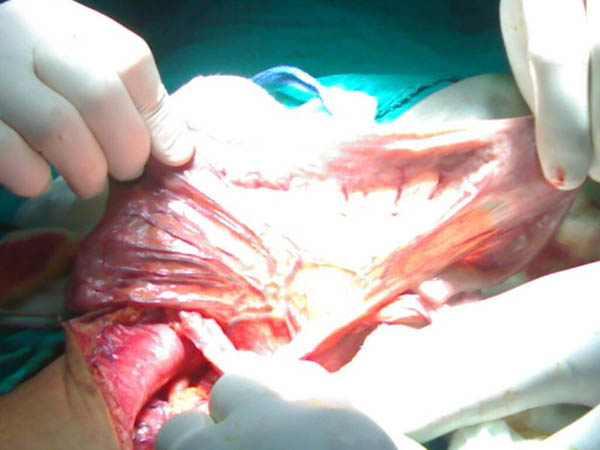
**A photograph showing the whole length of small gut completely free from the already described pneumatosis cystoides intestinalis**.

Laparotomy, through the previous midline incision, was done and exploration was systematically performed and denoted hugely distended stomach with cicatrisation at the site of previous closure of perforated duodenal ulcer and the whole length of small gut was completely free from the already described pneumatosis cystoides intestinalis (Figure 2). Gastro-jejunostomy with truncal vagotomy were done to bypass the outlet obstruction.

## Discussion

Pneumatosis cystoides intestinalis is a rare disease and the exact cause is probably a combination of associated diseases causing elevated pressure and mucosal damage allowing gas-forming microorganisms to enter the bowel wall, thus forming the cysts [9].

The author in this case report put the question whether pneumatosis cystoides intestinalis is the direct cause of perforated duodenal ulcer or the result of this disease process as advocated in other pervious reports [10,11].

Previous data stated that the cysts may be located in the subserosa, submucosa, and, rarely, the muscularis layer [1,4]. They may be single or multiple and vary in size from microscopic to several centimeters in diameter [4]. They are usually lined by mixed inflammatory cells, macrophages, or foreign body giant cells [12]. In the present case, the author found that the cysts were completely located in the subserous plane with varying sizes and shapes.

Usually, no treatment is necessary for 85% of patients who are asymptomatic. The resolution of gas collections has been reported after inhalation of 70% of oxygen and after hyperbaric oxygen therapy [4,9,13] Good results can be achieved in most cases by conservative means, but surgical treatment may be necessary in some cases [13]. Surgery should be avoided unless there are signs of severe inflammation, metabolic acidosis or portal venous gas, which are indicators of more serious diseases [13,14].

In the post-operative period, the patient of the present case was given metronidazole 500 mg / 12 hour as intravenous infusion together with cefotaxim 1 gm/ 8 hour. We noticed that the cysts showed complete resolution during the second operation with no adhesion formation or amalgamation between the intestinal loops. This finding came in agreement with those data reporting that therapy with metronidazole up to 1500 mg daily was effective to cause resolution of pneumatosis cystoides intestinalis [15]-[17].

## Conclusion

The pneumatosis cystoides intestinalis is a rare disease and suspicion of this disease process should be based on imaging and clinical finding. The therapy can be conservative or surgical in restricted situations.

## Consent

Written informed consent was obtained from the patient for publication of this case report and accompanying images. A copy of the written consent is available for review by the Editor-in-Chief of this journal.

## Competing interests

The author declares that he has no competing interests.

## See Additional file 1

## Supplementary Material

Additional file 1 All cysts were intact. Click here for file
